# Optimization of chemical conditions for metabolites production by *Ganoderma lucidum* using response surface methodology and investigation of antimicrobial as well as anticancer activities

**DOI:** 10.3389/fmicb.2023.1280405

**Published:** 2024-01-22

**Authors:** Alireza Tajik, Hamid Reza Samadlouie, Amir Salek Farrokhi, Amir Ghasemi

**Affiliations:** ^1^Department of Food Science and Technology, Faculty of Agriculture, Shahrood University of Technology, Shahrood, Iran; ^2^Department of Immunology, Pasteur Institute of Iran, Tehran, Iran

**Keywords:** *Ganoderma lucidum*, optimization, nanoparticles, polysaccharide, ganoderic acid, antimicrobial activity

## Abstract

*Ganoderma lucidum* (*G. lucidum*) is a medicinal mushroom that is known for its ability to produce compounds with physiological effects on human health. This research was undertaken to amplify the production of bioactive components of *G. lucidum* under optimal cultivation conditions, obtained in a submerged state and utilized in solid state fermentation, with the purpose of enhancing antimicrobial and anticancer activities. The results indicated that titanium dioxide (TiO_2_ NPs), magnesium oxide nanoparticles (MgO_2_ NPs), and B6, along with glucose syrup and CLS syrups, were the most effective for producing GA, while wheat starch and whey protein, along with MgO_2_ NPs and B6 vitamin, stimulated polysaccharide production using the One Factor at a Time (OFAT) method. After screening, the response surface method (RSM) statistically indicated that the media containing 42.11 g/L wheat starch with 22 g/L whey protein and 50 g/L glucose syrup with 30 g/L CSL were found to be the best conditions for polysaccharide (21.47% of dry weight biomass) and GA (20.35 mg/g dry weight biomass) production, respectively. The moss of the fruit body of *G. lucidum* produced under optimal GA conditions had the highest diversity in flavonoids and phenolic acids and significant antimicrobial activity against *Esherichia coli* (*E. coli*) and *Bacillus subtilis* (*B. subtilis*). In addition, the IC50 levels of shell and stem of *G. lucidum* were 465.3 and 485.7 μg/mL, respectively, while the moss did not reach 50% inhibition. In the end, the statistical approaches utilized in this research to elevate the levels of bioactive components in the fruiting body of *G. lucidum* produced a promising natural source of antimicrobial and anticancer agents.

## Introduction

Medical mushrooms as main producers of bioactive components with impressive effects on health promotion are a major area of interest within the field of biotechnology and pharmacology (Pan et al., 2022). Among various mushroom, *G. lucidum* has received considerable critical attention for its tendency to produce high-value bioactive components such as polysaccharide, many oxygenated triterpenes (especially ganoderic acid) and phenolic components with enormous medicinal properties such as anticancerous, antimicrobial and antitumor activates (Ahmad et al., 2022; Yang et al., 2022). In the past 2 decades, a number of researchers have sought to determine the GA, polysaccharide, and phenolic components as secondary metabolites of *G. lucidum* cultivated under the different formulated deciduous woods (Heleno et al., 2012; Ma et al., 2019; Lin et al., 2022; Liu et al., 2022). Physicochemical conditions of solid state fermentation found to be influencing *G. lucidum* metabolites have been explored in several studies as the formulated medium is so similar to natural habitat (Murugesan et al., 2007). Despite its efficacy, such fermentation method suffers from several major drawbacks: Dependence on the culture medium employed for secondary metabolite production and relatively long time of cultivation to harvest the fruit at the optimum stage of maturity (Fang and Zhong, 2002b). Most importantly, the production of secondary metabolites by *G. lucidum* in solid state fermentation is heavily influenced by the key substrates used in formulated media. As a primary substrate in solid state, starch plays a crucial role in promoting the production of secondary metabolites by *G. lucidum* (Zhou et al., 2012; Berovic and Zhong, 2023). Formulating a solid state media for mushroom cultivation relies heavily on the expertise of cultivators, as no statistical analysis has been conducted on solid state mediums to determine the most effective substrate for promoting *G. lucidum* growth and metabolites. The lack of precise measurements of biomass and metabolites in the solid state fermentation before the fruiting process has hindered the optimization process (Torres-Mancera et al., 2018). Previous research comparing submerge and solid state fermentation has found submerge fermentation is of series advantages over solid state such as the easy control of the physical and chemical conditions of media, the relatively lower spore contamination and cost-effective fermentation method. There is a consensus among biotechnology research scientists that the quality and quantity of macro elements such as carbon, nitrogen, and mineral elements substrates considerably affect the mushroom proliferation and secondary metabolites synthesis in submerge fermentation (Samadlouie et al., 2018). In the same vein, nanoparticles have several properties that are ideally suited to stimulate the rate of microbiological reaction (Gonabadi et al., 2021; Dong et al., 2022). Likewise, vitamins, especially the B group, as cofactor compounds, which are required in very small amounts, play a vital role in microbial growth and metabolite productions (LeBlanc et al., 2013; Miret and Munné-Bosch, 2014). A large and growing body of literature has investigated to identify the appropriate statistical method(s) for optimization of the physico-chemical conditions of medium for metabolite productions. Efficient productivity of polysaccharide and GA can be accomplished via appropriate key substrate selections and subsequently optimization methods (Ma et al., 2013). Among various statistical methods, response surface methodology (RSM) has been worldwide employed to improve the mathematics achievement of optimum conditions for key substrates affecting the responses (Malinowska et al., 2009; Ma et al., 2013). Before the optimization, key substrates with high positive impacts on responses should be detected by screening method like one a factor at a time method (Singh et al., 2011). Notably, studies on the effect of titanium dioxide (TiO_2_ NPs), magnesium oxide nanoparticles (MgO_2_ NPs), and vitamin B6 on secondary metabolites production in submerge conditions are scare, hence, the study provides recent updates on using these substrates in the formulated media. More importantly, the phenolic components of various parts of *G. lucidum* fruitbody, moss, stem and shell, have not been well-characterized yet. Therefore, the objective of this research was the optimization of *G. lucidum*’ polysaccharide and GA production in submerge fermentation, and finally, the optimal condition was applied in solid state and then the GA, total phenol as well as phenolic components profile of the mushroom fruit-body were characterized, following that the antimicrobial and anticancer activates of the extractions of different parts of *G. lucidum* fruit were examined.

## Materials and methods

### Materials and chemicals

*Ganoderma lucidum* (Sp. GIRAN17) was obtained from Faculty of Biological Sciences, Shahid Beheshti University, Tehran, Iran. Almost all materials were purchased from Merck Company. Concentrations were obtained under low pressure in a rotary evaporator (Heidolph Laborota 4000 efficient rotary evaporator, Germany). The extracts were dried by vacuum freeze-drying (Christ Alpha 1–2 freeze-dryer). TiO_2_ NPs and MgO_2_ NPs were attained from United States Research Nanomaterials Inc. with a purity of ±99% and an average size of 15 and 20 nm, respectively.

### Microorganisms, inoculums, and cultivation conditions

The strain was grown on potato dextrose agar (PDA), and for long-term preservation, it was maintained at a low temperature at 4°C. 250 mL Erlenmeyer flasks with 50 mL formulated medium was used to provide sufficient oxygen supply for fungi growth and metabolites production. 30 g/L glucose and 10 g/L yeast extract with macro mineral elements was served in 5 day fermentation for inoculum development to obtain maximum cell viability and concentrations. 5 percent of fresh seed culture was added to the fermentation media for growth efficiency and stimulation of secondary metabolite production (Esfandiyari Mehni et al., 2022).

### Fermentation media

Various substrates such as carbon (Corn and wheat starch, glucose powder, glucose, and fructose syrup) and protein [Corn Steep Liquor (CSL), Yeast extract, meat peptone, Fat-free soya flour, and Sweet whey powder] as macro elements substrate for evaluation of their effects on the production of polysaccharide and ganoderic acid were utilized. MgSo_4_. 7 H_2_0 (1 g/L), CaCl_2_ (0.2 g/L), KH_2_Po_4_ (1.5 g/L), and CaCo_3_ (1.5 g/L), a rotary shaker at 185 RPM and 30°C as constant factors were chosen (Xu et al., 2008). In a pioneering approach to improve the production of secondary metabolites, the effect of TiO_2_ NPs and MgO_2_ NPs in three concentrations (0.1, 0.05, and 0.01 g/L) as well as vitamin pyridoxine (B6) in four concentrations (μl) of 100, 200, 1,000, and 3,000 on the growth and formation of secondary metabolites (GA and polysaccharide) of *G. lucidum* in liquid culture medium were investigated.

### Analytical methods

#### Measurement of GA

Dry biomass of *G. lucidum* was used for GA extraction with 70% (v/v) ethanol and determined as previously described (Fang et al., 2002). Briefly, 10 mL of 70% (v/v) ethanol was served to extract GA from 0.1 g mycelium powder. Centrifugal separation was performed to separate the impurities, and then the supernatants were dried at 50°C under rotary evaporation. 5 mL chloroform was added to extract GA from the suspension of residues dissolved in 5 mL of water. After that, chloroform was removed and 5% (w/v) NaHCO_3_ with pH 3 was mixed with water to extract the desired components. Chloroform was added again to extract GA from the NaHCO_3_ solution and a relatively pure solution of GA in chloroform was dried using rotary evaporation at 40°C. The obtained precipitate was dissolved in anhydrous ethanol and the GA content was detected by spectrometric method at 245 nm wavelength. Thymol (Merck, 108,167, crystal) and absolute ethanol were used as a standard and blank solution. The concentrations of calibration thymol solutions ranged from 0.1 to 1 mg/mL.

#### Isolation and purification of polysaccharide

*Ganoderma lucidum* biomass was centrifuged (12,000 *g*, 5 min) to sediment from the formulated medium, and then distilled water (two times) was used for washing extracted biomass. Dry and crushed biomass was utilized to extract polysaccharides. The biomass was heated three times for 3 h at the boiling temperature of 96% ethanol solution to remove fats, coloring agent, free small molecules, and oligosaccharides. Polysaccharide of dry biomass was extracted thrice with distilled water at 70°C for 3 h. The supernatant was separated and purified by centrifuging at 3,000 rpm. The solution was deproteinated by sevag method (1-butanol: chloroform at a ratio of 1:4, v/v; Staub, 1965). The sevag reagent was removed and a vacuum rotary evaporator (Heidolph Laborota, Germany) was employed to remove 2/3 water phase (supernatant), and then dialyzed against deionized water for 48 h. The concentrated water-soluble polysaccharides were precipitated by using 96% ethanol maintained at 4°C for 24 h. The precipitated polysaccharides were collected by centrifugation, and a vacuum Alpha 1–2 freeze-dryer (Christ, Germany) was used to attain crude *G. lucidum* polysaccharides (CGLP; Samadlouie et al., 2020).

#### Preparation of *Ganoderma lucidum* solid state medium for fruit production

The optimized medium obtained by statistical methods was served to prepare the solid state medium for fruit production by *G. lucidum*. Beech wood sawdust and chips in equal amount was served as a basic substrate, detoxified by alkaline solution (NaOH) and formulated with glucose and CLS syrup. As these substrates were of a protective impact on spore contamination, naturally presented in Beech wood, a concentration of 50 ppm of NaOCl was used which had a detrimental effect on heat resistant spores (data not shown) and soaked the mixed substrates for 6 h and then sterilized at 121°C for 2.5 h. Seeds were added to a sterilized medium and incubated at appropriate conditions (Stamets, 2011) to harvest *G. lucidum* fruits.

#### Extraction of *Ganoderma lucidum* dry matter, total phenolic content, and analysis of phenolic compounds

Dried and fine crushed moss, stem and shell of *G. lucidum* were extracted with 90% ethanol (2,000 mL) at 25°C for 72 h. The extracts were centrifuged and then concentrated by a vacuum rotary evaporator (Heidolph Laborota, Germany; Nickavar and Esbati, 2012). The total phenolic contents (TPCs) were measured by Folin–Ciocalteu reagent and gallic acid (GAE) standard. Briefly, GAE standard dissolved in absolute ethanol was mixed with Folin Ciocalteu reagent. After 10 min, sodium carbonate solution (75 mg/mL) was mixed with the standards solution and maintained for 30 min at ambient temperature. UV–Vis spectrophotometer was employed to measure the absorbance of mixed solution at 765 nm. Exactly the same went for dry extracted ethanol solution of *G. lucidum* and the TPC was reported as mg GAE per gram of the ganoderma dry tissue (Nickavar and Esbati, 2012). *Ganoderma lucidum* dry biomass was used to extract phenolic components and phenolic profiles were determined as previously stated (Yahia et al., 2017).

The structure of the phenolic components was examined by an agilent 6410 QqQ, equipped with an Electrospray Ionization interface (ESI), with the following settings: Drying gas temperature (nitrogen): 350°C, drying gas flow rate: 9 L/min, nebulizer pressure: 40 psig, Injection Volume: 25 μL, skimmer: 60 V, fragmentor voltage: 220 V, capillary voltage: 3,500 V, and scan range of m/z: 50–1,000.

#### Antimicrobial activity of *Ganoderma lucidum*

The antibacterial activity of the extractions of the various parts of *G. lucidum*, shell, stem, and moss, against *E. coli* (ATCC25922) and *B. subtilis* (PTCC1715), was determined by the broth microdilution technique. Briefly, the bacteria were incubated overnight at 37°C in MHB (Mueller Hinton broth). 100 μL of the extractions of the various part of *G. lucidum* at the following concentrations: 62.5, 125, 250, 500, and 1,000 μg/mL, with 100 μL of bacteria were mixed in 96 well microplates, to reach 5 × 10^5^ colony-forming units (CFU)/mL of the examined bacteria. Erythromycin at a concentration of 32 μg/mL was used as positive control and 5 × 10^5^ CFU/mL of examined bacteria was served as growth control. All media was incubated at 37°C for 24 h. 20 μL of 0.5% solution (g/L) 2,3,5-triphenyl-tetrazolium chloride was added to each well after 22 h, following that 2 more hours of incubation was applied to determine microbial growth and MIC. The colorless samples were incubated in Brain Heart Infusion (BHI) agar and the lowest concentration that totally eradicated microbial growth was considered MBC (Valgas et al., 2007).

#### Cell culture and viability analysis

Breast cancer cell line MDA-MB-231 was purchased from National Cell Bank of Institute Pasteur of Iran. Dulbecco’s Modified Eagle Medium (DMEM), supplemented with 1% penicillin/streptomycin and 10% heat-inactivated fetal bovine serum was served to culture MDA-MB-231 cells line at 37°C with 5% CO_2_. To achieve high cellular viability, cells were subcultured with trypsin at the near confluency, following that 1 million cells were reseeded into a fresh T-75. MTT assay was served to determine cell viability of MDA-MB-231 and IC50 values (half maximal inhibitory concentration) of stem, shell, and moss of *G. lucidum* against MDA-MB-231. 10,000 cells per well were incubated and exposed to *G. lucidum* extractions for 48 h at 37°C in a 96-well plate. Serial dilutions of *G. lucidum* extractions 0, 62.5, 125, 250, 500, and 1,000 μg/mL were added into each of 96-well plates containing 10,000 cells. Cytotoxic evaluations of the extractions against MDA-MB-231 cancer cell line were evaluated using MTT assay according to the previous research (Hastings and Kenealey, 2017).

### Statistical method

One Factor at a Time method was served to identify the key substrates among the various substrates which exerted high influences on GA and CGLP production, and then the selected substrates were optimized using response surface method. Central composite design (CCD), a common form of response surface methodology (RSM) in statistical package Design-Expert, version 7.0.0, Stat-Ease, Inc. Minneapolis, MN, United States, was applied to design the experiments and statistically optimize the quantity of substrates influencing the responses. The ANOVA was used to verify the significance of the models, substrates, and their interactions on the responses. The least squares method was used to formulate second equation. All the experiments were conducted in triplicates. The means and standard deviations (SD) were obtained by Microsoft Excel (2023) software. The SPSS 16.0 software was used to compare the differences among means at the level of 0.05 by Duncan’s multiple range test.

## Results and discussion

### Effect of carbon sources on biomass growth rate, CGLP, and GA productions

**Figure 1:**
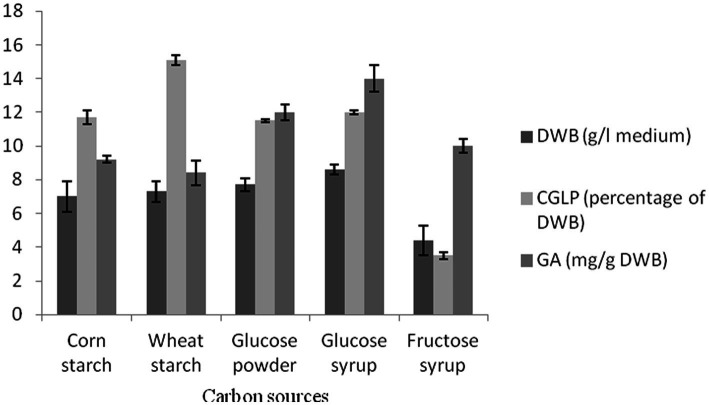
The effect of various carbon sources on DWB (percentage of medium), CGLP (percentage of DWB), and GA (mg/g DWB). The results are the mean ± SD of three independent experiments.

Carbohydrates have been identified as the main contributing factors for the *G. lucidum* growth and polysaccharide production (Hsieh et al., 2005). Simple (glucose powder, glucose syrup, and fructose syrup) and complex (corn and wheat) carbohydrates were served to evaluate their effects on biomass dry weight (BDW), CGLP, and GA productions. The highest biomass dry weight (BDW) was accumulated with glucose syrup (0.86 percentage of medium), while the lowest was accumulated in fructose substrate (0.44 percentage of medium). The results indicated that there was no significant difference between corn and wheat starch. The unit of single simple sugars such as glucose substrate found to be influencing biomass production have been explored in several studies (Xu et al., 2008; Hu et al., 2018). In term of CGLP production, the medium containing wheat starch and fructose syrup had the highest (15.1 percent of DWB) and lowest (3.5 percent of DWB) CGLP, respectively. No significant difference was observed between the three carbon sources of corn starch, glucose powder, and glucose syrup. According to recent studies, the suitability of wheat starch compared to other carbohydrate sources may be a result of the potential for these substrates to be quickly transformed into accumulated polysaccharide (Fan et al., 2007; Maalej et al., 2014). As shown in Figure 1, the highest GA was obtained in the medium containing glucose syrup. Following that, glucose powder showed the highest (12 mg/g DWB) GA. Other carbon sources supported lower GA productions. These results were supported by Wei et al. (2016) who reported that glucose is the preferred carbon source for biomass and total GA production (Wei et al., 2016). wheat starch and glucose syrup turned out at the top in impacting CGLP and GA productions, respectively, which were utilized separately to further study its effect on metabolite productions with using different substrates employed.

### Effect of nitrogen source on DWB, CGLP, and GA productions

**Figure 2:**
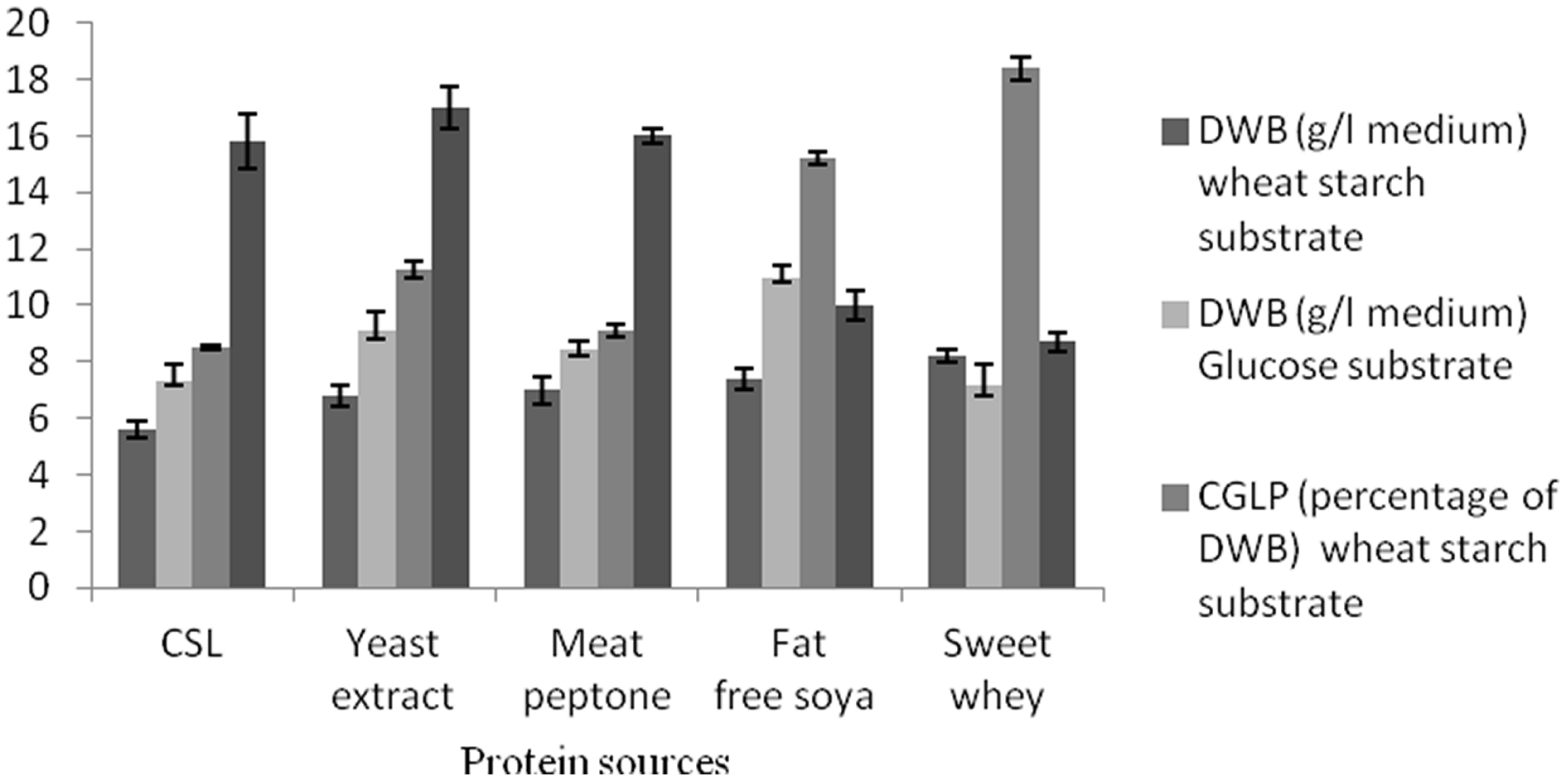
The effect of various protein sources on DWB (percentage of medium), CGLP (percentage of DWB), and GA (mg/g DWB).

As the main substrates involved in the structure of microbial biomass and enzyme production, proteins have been successfully used as a vital macronutrient in medium to stimulate microbial growth and metabolic production (Esfandiyari Mehni et al., 2022). Two media with different carbon sources, wheat starch, and glucose syrup, were used to investigate the effects of various nitrogen sources on DWB, CGLP, and GA productions. The analysis showed that the best candidate for achieving the greatest DWB was obtained in the medium that comprised of whey powder substrate, succeeding that soybean powder. However, the lowest DWB were accumulated in media containing CSL syrup nitrogen source. No significant differences were observed between meat peptone and yeast extract in biomass production. As whey protein is rich in essential amino acids, lactose and micronutrients (Patel, 2015), it is considered as the best macronutrient substrate for microbial growth. In terms of CGLP production, whey protein was the most potent, with soybean powder substrate coming in second, and CSL syrup substrate producing the least (8.5 percent of DWB) CGLP content in wheat starch medium. Detailed examination of whey protein by Song et al. (2007) and Lee et al. (2003) showed that such a protein source could be a suitable substrate for mycelia and polysaccharide productions (Lee et al., 2003; Song et al., 2007). As shown in Figure 2, culture medium containing yeast extract, meat peptone and CSL stimulated the most (17 mg/g DWB) GA formation. On the other hand, the lowest average GA was found in medium containing whey substrate and glucose syrup medium. Overall, a reasonable amount of GA was produced with CSL syrup, as this protein source is the most cost effective, and was therefore selected for further studies. No previous study has investigated the effect of CSL syrup on GA formation by *G. lucidum.*

### Effect of initial pH on biomass growth rate, CGLP, and GA productions

**Figure 3:**
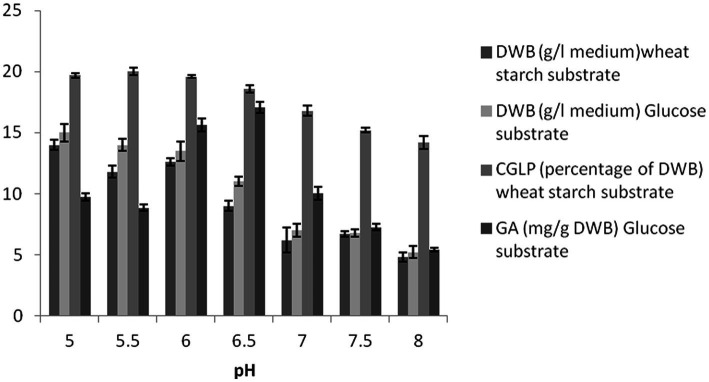
The effect of pH on DWB (percentage of medium), CGLP (percentage of DWB), and GA (mg/g DWB).

At pH 5, the maximum DWB was obtained under the optimal conditions resulting from the OFAT method. The data show an inverse correlation between the higher initial pH and average DWB in the two formulated media, indicating that *G. lucidum* biomass tends to accumulate in slightly acidic conditions. The amount of CGLP at initial pH between 5 and 6.5 was almost close to each other and thus suitable for CGLP production. Any increase in pH toward a higher level negatively affects the CGLP content (Figure 3). Similarly, Supramani et al. (2019) showed that lowering the initial pH from 6 to 4 stimulated biomass and CGLP production. In term of GA production, medium with initial pH 6.5 and 8 had the highest (17.06 mg/g DWB) and lowest (5.41 mg/g DWB) GA, respectively. This view was supported by Fang and Zhong (2002a) who stated that maximum GA production was achieved at an initial pH of 6.5.

### Effect of TiO_2_ NPs on DWB, CGLP, and GA productions

**Figure 4:**
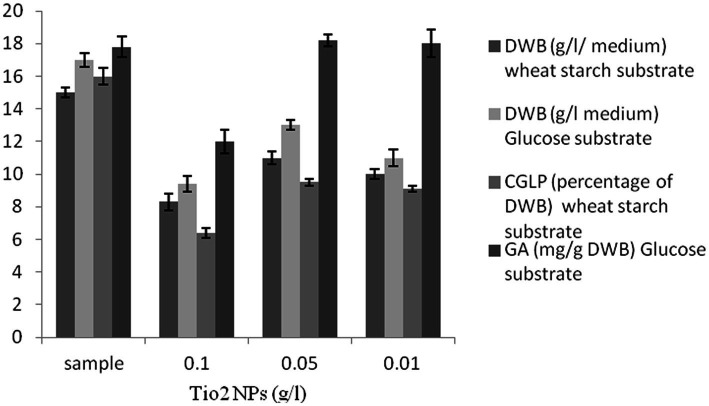
The effect of various concentrations of TiO_2_ NPs (g/L) on DWB (percentage of medium), CGLP (percentage of DWB), and GA (mg/g DWB).

Titanium dioxide nanoparticles negatively impacted biomass production. There was a strong negative correlation between the concentration of titanium dioxide nanoparticles and DWB. In the last 2 decades, a number of researchers have demonstrated the antimicrobial activity of titanium dioxide nanoparticles (Mesgari et al., 2021). As far as CGLP production was concerned, the control sample showed the highest internal CGLP formation (16 percent of DWB). TiO_2_ NPs had a negative effect on the formation of CGLP. As shown in Figure 4, the culture supplemented with 0.05 g/L titanium dioxide had the highest GA content (18.2 mg/g DWB). It can be concluded that the antimicrobial activity of TiO_2_ NPs (de Dicastillo et al., 2020) stimulated GA production. The stimulatory effect of TiO_2_ NPs on the GA production as a secondary metabolite could be strongly attributed to the chemical defense of cells already exposed to harmful components like nanoparticles. In the same vein, Gu et al. (2018) in their review article reported that the biosynthesis of GA in *G. lucidum* was triggered by stressors (Gu et al., 2018), so 0.05 g/L titanium dioxide was used in the experiment to optimize the carbon to nitrogen level for GA production.

### The effect of MgO_2_ NPs on DWB, CGLP, and GA productions

**Figure 5:**
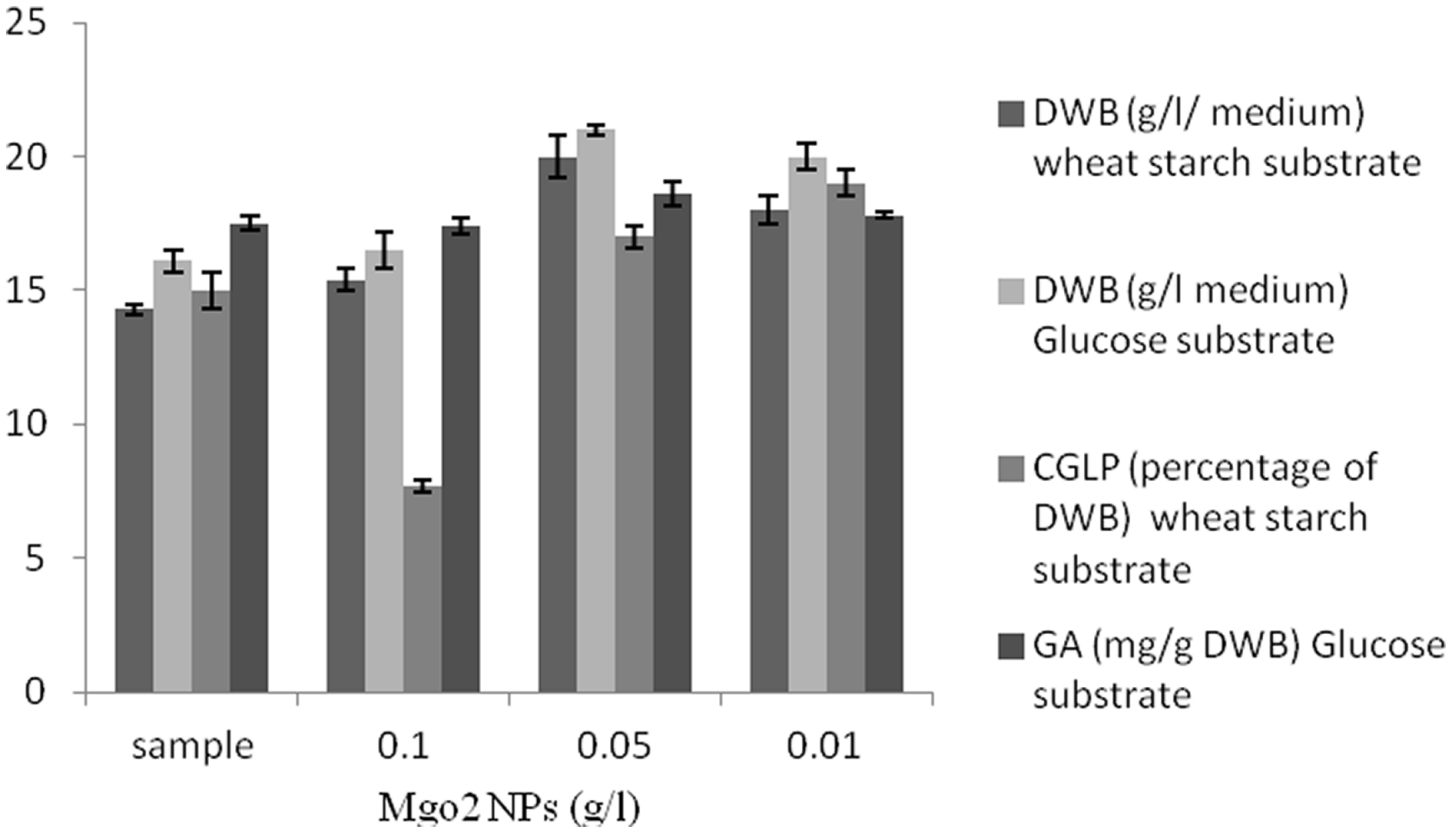
The effect of various concentration of magnesium oxide nanoparticles (g/L) on DWB (percentage of medium), CGLP (percentage of DWB), and GA (mg/g DWB).

As shown in Figure 5, MgO_2_ NPs had positive impacts on DWB in all concentrations compared to the control sample. Importantly, maximum CGLP was produced with 0.01 g/L nanoparticles of magnesium oxide, followed by 0.05 g/L of MgO_2_ NPs. The amount of CGLP in the control sample was higher than that of the medium containing 0.1 g/L MgO_2_ NPs. In general, the highest concentration of MgO_2_ NPs adversely affected the formation of CGLP. Therefore, in the experiment to optimize the ratio of carbon to nitrogen to produce the maximum internal CGLP of biomass, a concentration of 0.01 g/L of MgO_2_ NPs was considered for further studies. As shown in Figure 5, 0.05 g/L MgO_2_ NPs was the best concentration of nanoparticles to stimulate GA (18.6 mg/g DWB). No significant difference was observed between the medium containing 0.1 g/L magnesium oxide nanoparticles and the control sample. Various factors have influenced the toxicity of nanoparticles on microorganisms, including the characteristics of nanoparticles, species of microorganisms, physicochemical conditions of the media and its concentration (Pareek et al., 2018). Therefore, in the experiment of optimizing the carbon to nitrogen ratio to produce the maximum GA of biomass, a concentration of 0.05 g/L of MgO_2_ NPs was utilized.

### Effect of vitamin pyridoxine on DWB, CGLP, and GA productions

**Figure 6:**
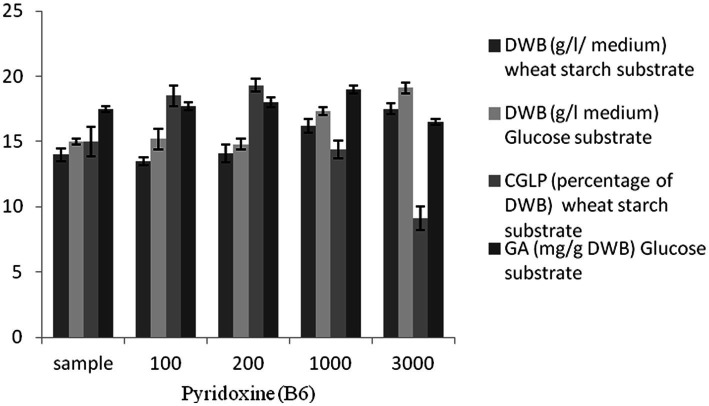
The effect of various concentration of pyridoxine (B6) μL on DWB (percentage of medium), CGLP (percentage of DWB), and GA (mg/g DWB).

There is a growing trend toward the use of micronutrients such as vitamins, which have significant effects on the effectiveness and efficiency of fermentation success. Pyridoxine exerts a considerable influence on carbon utilization and therefore promotes microbial growth and metabolites (Du Preez et al., 1985). Several studies were demonstrated that pyridoxin stimulated growth and secondary metabolites (Yang et al., 2008; Tripathi et al., 2012), however what is not yet clear is the impact of pyridoxine present and its concentration in the media on the growth and metabolites of *G. lucidum*. Based on this, pyridoxine at different concentrations was utilized to investigate its effect on microbial growth and secondary metabolites. A substantial increase in DWB (19.1 g/L in glucose syrup medium and 17.5 g/L in wheat starch medium) was observed at the maximum concentration of pyridoxine (3,000 μL). Maximum CGLP (19%) was attained at 200 μL pyridoxine. However, a further increase in pyridoxine concentrations was detrimental to CGLP production. As shown in Figure 6, 1,000 μL vitamin pyridoxine provoked the highest GA formation (19 mg/g DWB), while 3,000 μL vitamin pyridoxine resulted in the lowest GA content (16.5 mg/g DWB). In general, there was a positive correlation between vitamin pyridoxine concentration up to 1,000 μL and GA production, while a further increase in pyridoxine concentration up to 3,000 μL caused the formation of GA to decrease its lowest content. Therefore, vitamin pyridoxine with a concentration of 1,000 μL was considered in optimizing the carbon-nitrogen ratio to produce maximum GA content.

### Optimization of CGLP production using RSM

The main aim of this investigation was to optimize the amount of CGLP in DWB of *G. lucidum* in submerge fermentation. A wide range of amounts of wheat starch and whey powder, identified as key substrates by OFAT method, were optimized by CCD. OFAT indicated that pH 6.5, 0.05 g/L magnesium oxide nanoparticles and 200 μL of pyridoxine were suitable for CGLP production, selected as constant factors. The results of ANOVA showed that the linear, interaction and quadratic effects of whey powder (A) and wheat starch (B) on the amount of CGLP in liquid culture medium were significant (*p* < 0.05). As shown in Table 1, the *F* value of wheat starch was higher than that of whey powder. Wheat starch was found to be quantitatively superior to whey powder in the formation of CGLP.

**Table 1:** ANOVA for polysaccharide surface quadratic.

Source	Sum of squares	df	Mean square	*F* value	*p* value
					Prob > *F*
Model	40.26071	5	8.052143	17.2859	0.0082
A-whey	7.065748	1	7.065748	15.16836	0.0176
B-wheat	12.09222	1	12.09222	25.95891	0.0070
AB	0.7225	1	0.7225	1.551024	0.2810
A^2	17.49446	1	17.49446	37.55616	0.0036
B^2	11.07161	1	11.07161	23.76792	0.0082
Residual	1.863286	4	0.465821		
Lack of Fit	1.618286	3	0.539429	2.201749	0.4514
Pure Error	0.245	1	0.245		
Cor Total	42.124	9			
					

ANOVA was employed to determine a second order polynomial equation. The fitted second order polynomial equation of CGLP production at different concentrations of wheat starch and whey powder has demonstrated as Y is CGLP production, A and B are wheat starch and whey powder, respectively.


$$\begin{array}{l} Y=-8.79917+1.00398\times B+0.91336\times A-3.40000E\\ \;-003\times A\times B-0.019563\times B^{2}-9.96000E-003\times A^{2} \end{array}$$

The numerical value of the coefficient of determination (*R*^2^) for the CGLP production formula with non-significant lack of fit values (*p* > 0.05) was 0.9558; which indicates the degree of compliance of the data in the regression model (Table 1). From the numerical value of the coefficient of determination, it can be concluded that the regression models have been able to show and predict the relationship between the culture conditions (wheat starch and whey powder) and the formation of intracellular CGLP of the fungal biomass in the liquid culture medium. Hereupon, as the amount of wheat starch was increased from 25 g/L to 50, while keeping the nitrogen source of whey powder at a low concentration of 10 g/L, the quantity of CGLP climbed steeply from 15 to the highest value of 19 percentage of DWB (samples 4 and 5). On the other hand, when the quantity of whey powder was kept constant at the highest value of 30 g/L and the quantity of wheat starch was climbed up to the same quantity, the amount of CGLP formation started increasing at a lower rate from 17.7 up to 20 percent of DWB (sample 6 and 10). Such results indicated the adverse impact of high quantity of protein sources on increasing the rate of CGLP production (Table 2). Previous research has indicated that the higher content of nitrogen source has a negative impact on polysaccharide production (Hsieh et al., 2005).

**Table 2:** Results of CCD using two variables demonstrating observed polysaccharide.

Sample	Wheat starch g/L	Whey g/L	Actual polysaccharide percentage of DWB	Predicted polysaccharide
1	20	37.5	21.5	21.1
2	34.14214	37.5	18.3	18.5
3	5.857864	37.5	15.6	15.9
4	10	25	15	15
5	10	50	19	19.7
6	30	50	20	19.4
7	20	19.82233	16.5	16.3
8	20	37.5	20.8	21.1
9	20	55.17767	19	18.3
10	30	25	17.7	17.7

Moreover, the result showed that keeping whey powder constant at an average concentration of 20 g/L, when wheat starch climbed from 19.82 g/L up to 37.5, the formation of CGLP raised with an increase rate from 16.3 up to 21.1 percentage of DWB (sample 7 and 1) (Table 2).

Similarly, Hsieh et al. (2006) found that low nitrogen level stimulated polysaccharide production. The maximum rate of CGLP was reached when the quantity of wheat starch was headed toward 37.5 g/L, after which production started to irreversible decline at higher concentrations. This result was supported by Hsieh et al. (2006) and Supramani et al. (2019) who stated that higher concentration of carbon and nitrogen source were of a negative impacts on CGLP production by *G. lucidum*.

### CGLP production

**Figure 7:**
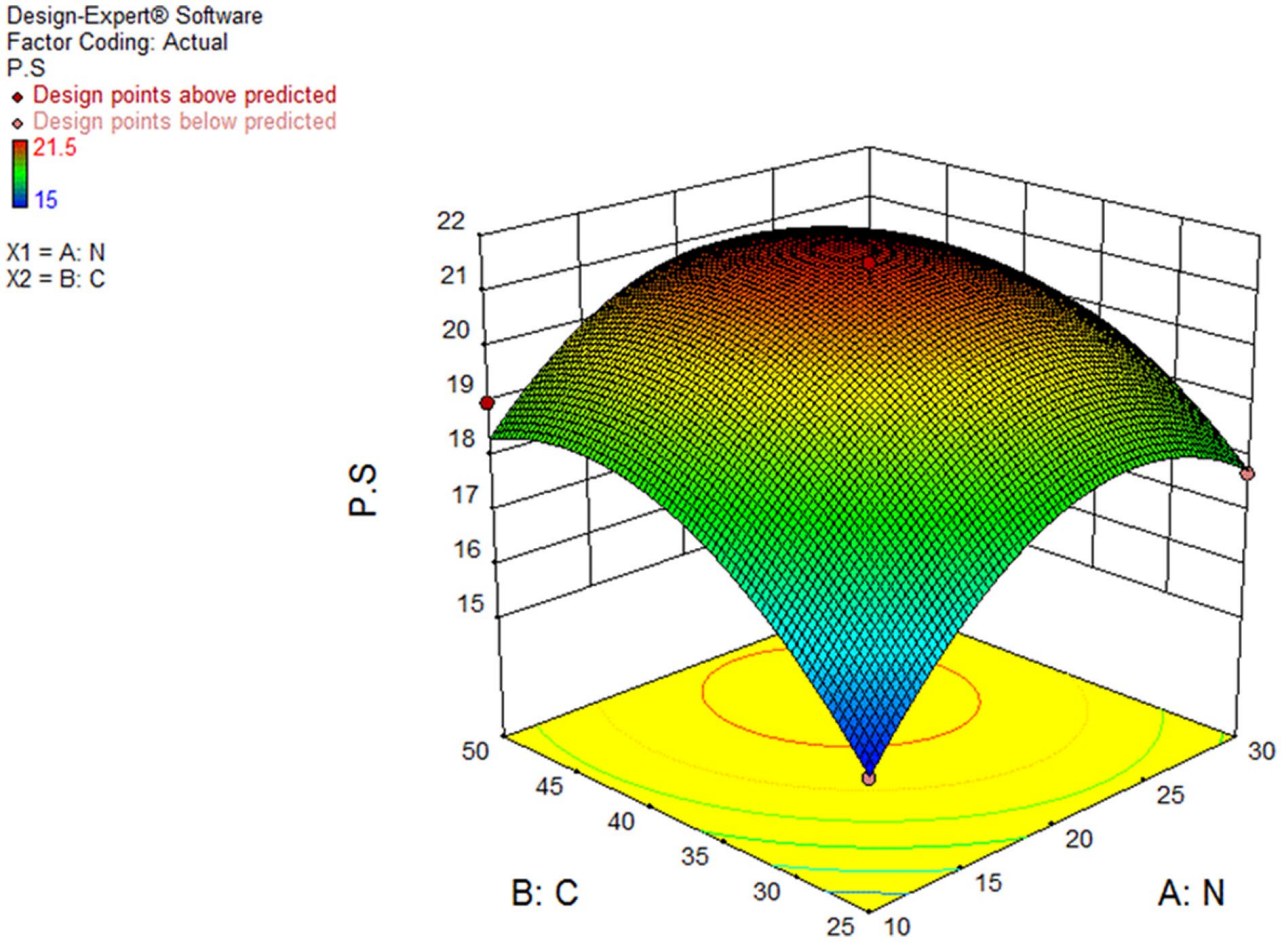
Curve of RSM for polysaccharide (PS) production by *Ganoderma lucidum* using wheat starch (C) and whey powder (N) as variable factors. PS (percentage of media), C and N (G/L).

Figure 7 demonstrated the effect of different levels of wheat starch and whey powder on CGLP production. As the response surface curve shows, the content of CGLP in DWB was stimulated by increasing the concentration up to middle content of both substrates in the liquid culture medium. The highest of CGLP content was obtained (21.47 percent of DWB) in medium containing (g/L) 42.11 wheat starch and 22 whey. The predicted value was implemented and the actual CGLP content of 22.5 percentage of DWB was achieved, significantly close to the result obtained by the RSM model. The lowest amount of CGLP, 15.04 (percentage of DWB), was observed in the medium containing 25 g/L of wheat starch and 10 g/L of whey protein.

### Optimization of GA production using RSM

A range of two key substrates were applied: glucose syrup as a carbon source and CSL syrup as a nitrogen source based on the results of OFAT. Then, the amount of these two substrates was optimized by RSM method. pH 6.5, 1,000 μL of vitamin pyridoxine, 0.05 g/L TiO_2_ and MgO_2_ NPs were suitable for GA production so these factors were selected as constant factors.

ANOVA was employed to indicate a second order polynomial equation (Table 3). The fitted second-order polynomial equation of GA accumulation over glucose syrup and CSL syrup was demonstrated as where Y was GA production and A and B were glucose syrup and CSL syrup, respectively.


Y=+125.56230+0.94196×A+1.02872×B

**Table 3:** ANOVA for linear model.

Source	Sum of squares	df	Mean square	*F* value	*p* value	
Model	19.56	2	9.78	31.00	0.0003	Significant
A-G.S	11.09	1	11.09	35.17	0.0006	
B-CSL	8.47	1	8.47	26.84	0.0013	
Residual	2.21	7	0.3154			
Lack of fit	2.20	6	0.3671	73.42	0.0891	Not significant
Pure error	0.0050	1	0.0050			
Cor. total	21.76	9				

The coefficients of the linear polynomial model were obtained after analysis with the software and eliminating nonsense terms using the Forward method in the software.

The numerical value of the determination coefficient (*R*^2^) for the formula of GA production is 0.89; which indicates the degree of compliance of the data in the regression model. From the numerical value of the coefficient of determination, it can be concluded that regression models have been able to show and predict the relationship between culture conditions (glucose syrup and CSL syrup) and GA accumulation.

As shown in Table 4, GA content was stimulated by keeping the nitrogen source of CSL syrup constant and increasing the glucose syrup concentration (samples 1 and 2). The same result was obtained when glucose syrup concentration was kept constant and CSL syrup was slightly increased (samples 1 and 6). Higher concentrations of glucose syrup and CSL syrup had the greatest effect on GA formation. It was found that quality and quantity of carbon, especially simple carbohydrate and nitrogen sources, effectively stimulated GA production (Xu et al., 2008; Hu et al., 2017).

**Table 4:** Results of CCD using two variables demonstrating observed GA.

Sample	Glucose syrup (g/L)	CSL (g/L)	GA (mg/g DWB)
1	50	10	18.9
2	25	10	16.2
3	37.5	20	17.9
4	37.5	20	18
5	37.5	34.14214	19.6
6	50	30	21
7	25	30	17.4
8	19.82233	20	17
9	37.5	5.857864	16.1
10	55.17767	20	19.2

### GA production

**Figure 8:**
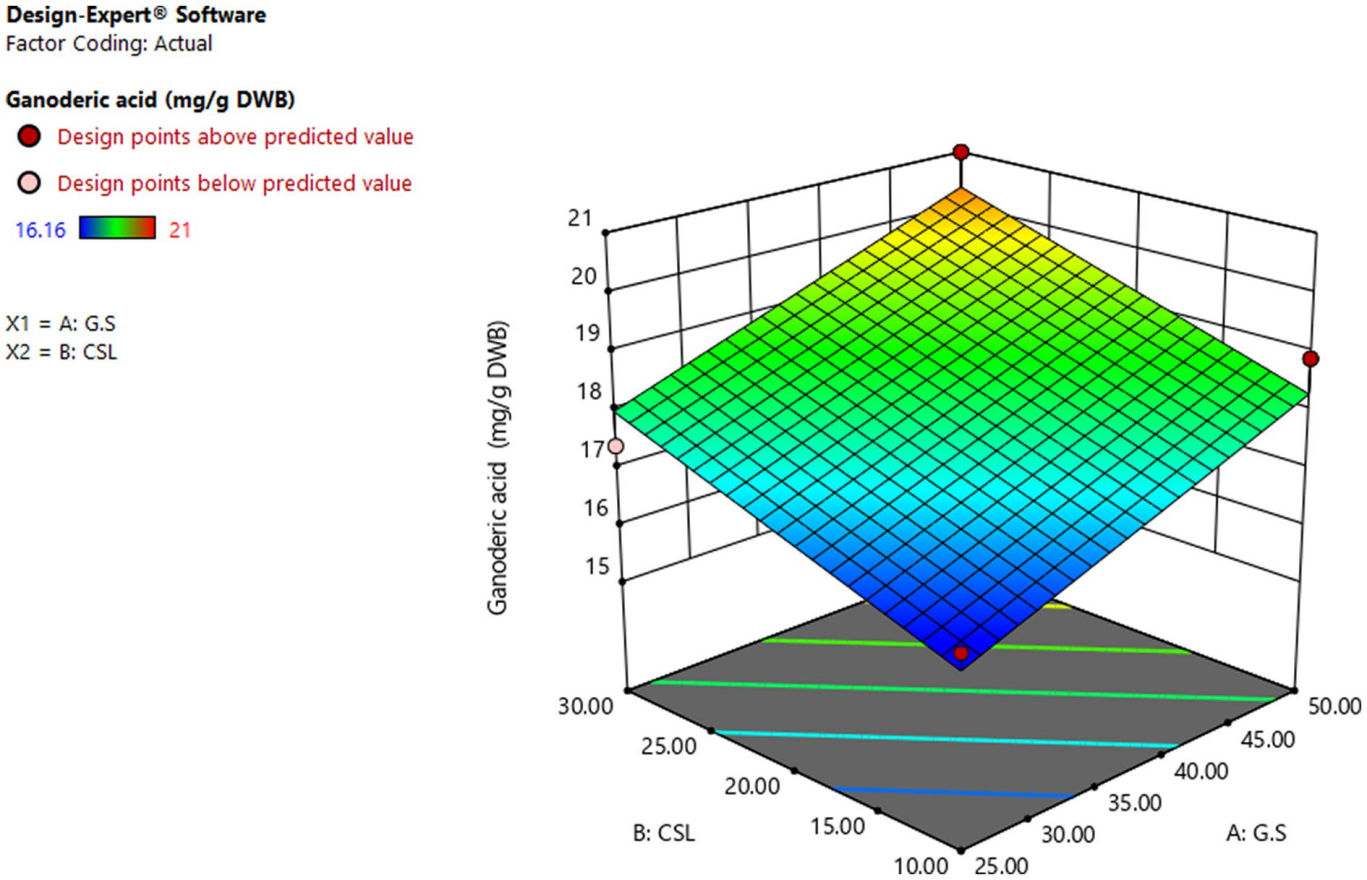
Curve of RSM for GA production by *Ganoderma lucidum* using glucose syrup (A) and CSL syrup (N) as variable factors.GA (μg/g DWB), C and N (g/L).

Figure 8 showed the effect of different levels of glucose syrup (B) and CSL syrup (A) on GA production. As the response surface curve showed, GA content in DWB was stimulated by increasing the concentration of both sources in the liquid culture medium. The highest GA content (20.35 mg/g DWB) was obtained in the medium containing 50 g/L glucose syrups and 30 g/L CSL syrups (Fang and Zhong, 2002b) supported the hypothesis that simple carbohydrate such as glucose at 50 g/L induced GA production. While the lowest content of GA, 16.08 mg/g DWB, was observed in the medium containing 25.92 g/L glucose syrup and 10.51 g/L CSL syrup. The predicted amount of selected substrates was run with the aim of producing maximum GA content, and the actual GA content was equal to 20 mg/g DWB, significantly close to the result obtained by the RSM model. Response surface methodology has been widespreadly used to optimize key substrates for the growth and metabolites production of fungi, but no research on glucose syrup and CLS syrup have been used to optimize GA production with such a statistical method. The lower production of GA in submerge fermentation compared to solid state fermentation (Tang and Zhong, 2002) was the main reason for applying optimal condition in solid state fermentation with basic media of Beech wood sawdust and chips.

### Solid state formulation toward *Ganoderma lucidum* fruiting

**Figure 9:**
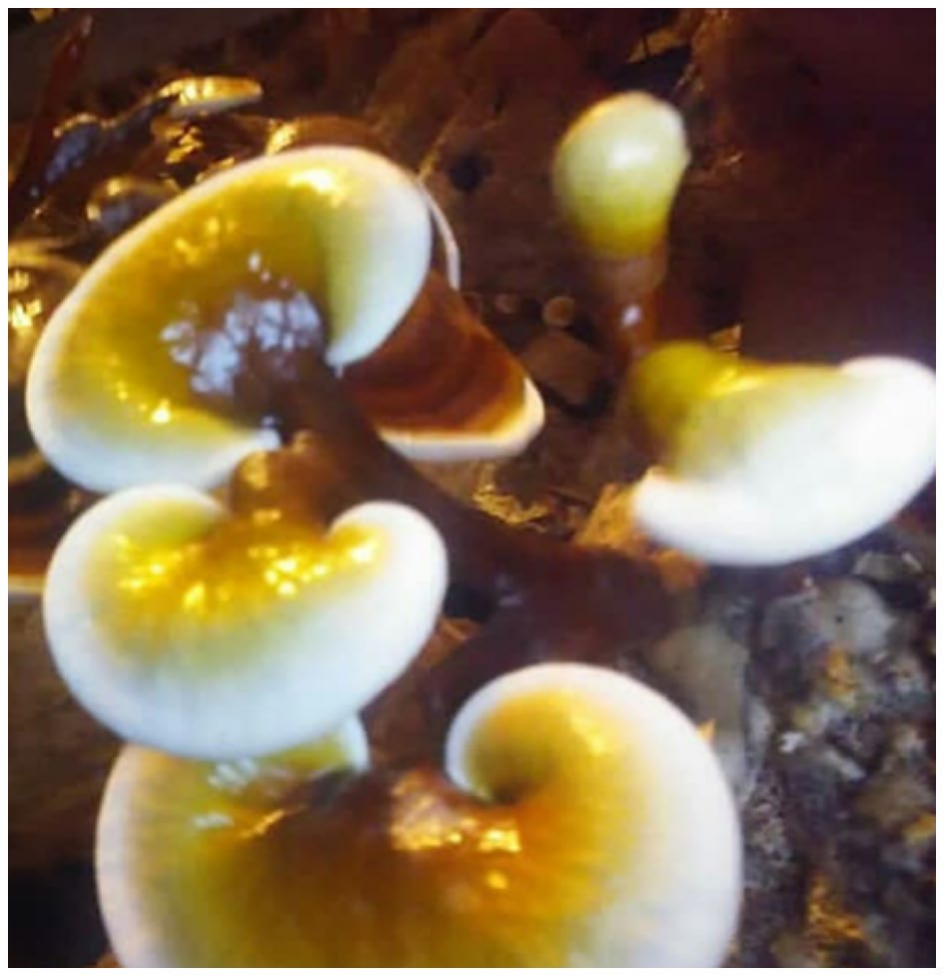
Fruiting body of *Ganoderma lucidum* cultivated under optimal condition predicted by RSM.

Optimum GA production condition was used to prepare solid state medium. Beech wood sawdust and chips in equal amount as basic medium was enriched with glucose and CLS syrup. Nutrient components added to wood chips had protective effects on heat-resistant spores, so in initial test, the formulated media were invaded by heat-resistant spores, despite the fact that the formulated medium was sterilized at 120°c for 2.5 h. To solve the problem of contamination and reduce the resistance of resistant spores before the sterilization process, all substrates were soaked in 50 ppm NaOCl for 6 h. Optimal condition for fruit production by *G. lucidum* was prepared based on book of Stamets (2011), and after 2 months all fruits were harvested (Figure 9). The optimal condition applied led to the production of maximum GA, 32.64 ± 0.5 mg/g.

The results indicated that solid state fermentation led to a significant increase in GA production compared to submerge fermentation (more than 63%: from 20 to 32.64 mg/g DWB). Dry matter, total phenolic component, and phenolic profiles of the moss, shell, and stem of the *G. lucidum* fruiting body were investigated. The results showed that solid state fermentation led to a significant increase in GA production compared to submerged fermentation (more than 63%: from 20 to 32.64 mg/g DWB). Dry matter, total phenolic components, and phenolic profiles of moss, shell, and stem of *G. lucidum* fruiting body were investigated.

### Total phenolic content and phenolic profiles

The dry matters of *G. lucidum* tissue extracted with 90% ethanol were between 6 and 11%, most of which was in shell (11%), followed by stems (10%) and then moss (6%), respectively. To measure the amount of phenolic components, the dried matters of *G. lucidum* tissue were dissolved in ethanol and brought to a concentration of 400 μg/mL. At this concentrations, 8.84 μg/mL total phenol was obtained in moss, 29.98 in shell and 30.26 in mushroom stems.

So that 2.21% of meat, 7.49% of skin, and 7.56% of mushroom stems were made up of phenolic compounds. These results indicated that the highest amount of phenolic compounds was obtained in the stem and the lowest amount was obtained in the fungal moss. Several attempts have been made to measure the content of *G. lucidum* phenolic compounds and its anioxidant activates. In these studies, conducted to determine the phenolic compounds of *G. lucidum*, it was discovered to be between 3.3 and 7.14/100 g GAE (Yuen and Gohel, 2005; Ferreira et al., 2009; Veljović et al., 2017).

What is not yet clear is the impact of various part of *G. lucidum* on phenolic content and their phenolic profiles. The ethanol extracts of *G. lucidum* phenolic profiles were determined by an Agilent 6410 QqQ, equipped with an Electrospray Ionization interface (ESI) and the results were showed in Table 5. The moss of *G. lucidum* fruit had the highest diversity variation in phenolic components, namely 16 phenolic compounds from the list of phenolic compounds, followed by 11 phenolic compounds in the skin and nine phenolic compounds in the stem. Previous studies have reported that phenolic acids (gallic acid and trans-cinnamic acid) and flavonoids (quercetin, kaempferol, hesperetin, and naringenin) were the major phenolic compounds identified in Ganoderma by Veljović et al. (2017). The present results showed that the moss, skin, and stem of *G. lucidum* fruit contained nine flavonoids and seven phenolic acids, seven flavonoids and three phenolic acids and five flavonoids and four phenolic acids, respectively. In the last 2 decades, a number of researchers have sought to determine the profile of phenolic acids and flavonoids of *G. lucidum* fruit (Karaman et al., 2010; Heleno et al., 2012; Saltarelli et al., 2015; Dong et al., 2019), However researchers have not addressed the phenolic acids profiles in various parts of *G. lucidum* fruit in much detail. Naringin and Myricetin and 3,4-Dihydroxybenzaldehyde were observed only in the shell of *G. lucidum* fruit. Protocatechuic acid, on the other hand, was the only phenolic acid found in the stem. Gallic acid, Kaempferol, Isoramnethin, Homogentisic acid, p-Coumaric acid, o-Coumaric acid, Quercetin, and Coumaric acid were specifically found in the fruit moss of *G. lucidum* (Table 5).

**Table 5:** Phenolic acids and flavonoids extracted from moss, shell and stem of *Ganoderma lucidum.*

Phenolic compounds	Polyphenol classes	Moss	Shell	Stem
1 Malic acid	Phenolic acids	+	+	+
2 Protocatechuic acid	Phenolic acids	−	−	+
3 Gallic acid	Phenolic acids	+	−	−
4 Kaempferol	Flavonoids	+	−	−
5 Isorhamnetin	Flavonoids	+	−	−
6 Sinapic acid	Phenolic acids	−	−	−
7 Pyrogallol	Other polyphenols	−	−	−
8 Homogentisic acid	Phenolic acids	+	−	−
9 5-Sulfosalicylic acid	Phenolic acids	−	−	−
10 Gentisic acid	Phenolic acids	−	−	−
11 p-Hydroxybenzoic acid	Phenolic acids	−	−	−
12 Catechin	Flavonoids	+	+	+
13 Chlorogenic acid	Phenolic acids	+	+	+
14 Vanillic acid	Phenolic acids	−	−	−
15 Caffeic acid	Phenolic acids	−	−	−
16 Syringic acid	Phenolic acids	−	−	−
17 Vanillin	Other polyphenols	−	−	−
18 Cinnamic acid	Phenolic acids	−	−	−
19 p-Coumaric acid	Phenolic acids	+	−	−
20 Ferulic acid	Phenolic acids	−	+	+
21 Veratric acid	Phenolic acids	−	−	−
22 Rutin	Flavonoids	+	+	+
23 Salicylic acid (2-Hydroxybenzoic acid)	Phenolic acids	−	−	−
24 Benzoic acid	Phenolic acids	−	−	−
25 Naringin	Flavonoids	−	+	−
26 o-Coumaric acid	Phenolic acids	+	−	−
27 Myricetin	Flavonoids	−	+	−
28 Resveratrol	Stilbenes	−	−	−
29 Quercetin	Flavonoids	+	−	−
30 Naringenin	Flavonoids	+	−	+
31 Hesperetin	Flavonoids	+	−	−
32 Formononetin	Flavonoids	−	+	+
33 Biochanin A	Flavonoids	+	+	−
34 Epicatechin	Flavonoids	+	+	+
35 Syringaldehyde	Other polyphenols	−	−	−
36 2,3,4-Trihydroxybenzoic acid	Phenolic acids	−	−	−
37 3,4-Dihydroxybenzoic aldehyde (Protocatechuic aldehyde)	Other polyphenols	−	+	−
38 m-Coumaric acid	Phenolic acids	+	−	−
39 Quercetin 3-D-galactoside	Flavonoids	−	−	−
40 Abscisic acid	hormone	+	+	+

### Antibacterial activates of ethanol extraction of *Ganoderma lucidum* shell, stem and moss against *Esherichia coli* (ATCC 25922) and *Bacillus subtilis* (PTCC 1715)

Antimicrobial potentiality of *G. lucidum* fruit body has long been of great interest in a wide range of biotechnology fields. In this research, six initial concentrations (μg/mL), 10,000, 5,000, 2,500, 1,250, 625, and 312 of *G. lucidum* shell, stem and moss were utilized to evaluate their antibacterial activity against *E. coli* and *B. subtilis* by McFarland standards. According to the results of Table 6, the MBC of shell, stem, and moss was 625, 625, and 312 μg/mL, respectively, which eradicated the entire population of *E. coli* after 48 h. At the concentration of 375 μg/mL shell, 500 μg/mL stem, and 250 μg/mL moss, only two colonies were observed, and these concentrations were considered as MIC, respectively. As far as *B. subtilis* was concerned, the phenolic extract at a concentration of 4,000 μg/mL shell, 4,000 μg/mL stem, and 3,000 μg/mL moss was able to kill the entire population of *B. subtilis*, even after 48 h no colony was observed. Only two colonies were observed at concentration of 3,000 μg/mL shell, 3,000 μg/mL stem, and 2,500 μg/mL moss, which were considered as MBC and MIC, respectively, (Table 5). The highest antibacterial activity with the lowest MIC and MBC of *G. lucidum* moss against *E. coli* and *B. subtilis* was most likely attributed to the greatest variation in the phenolic components of the moss compared to those of shell and stem. *Esherichia coli* as a germ negative Bacteria showed more susceptibility than *B. subtilis* at the lowest concentration of 312 μg/mL of *G. lucidum* moss. Data from several studies suggested that the fruiting body of *G.lucidum* exhibited inhibitory activity against *E. coli*, most importantly, the MIC were reported (mg/mL) 1.25 (Shah et al., 2014), 8 (Quereshi et al., 2010), and 15 (Mehta and Jandaik, 2012).

**Table 6:** The effect of various concentrations of stem, shell and moss of *Ganoderma lucidum* against *Esherichia coli* (ATCC25922), and *Bacillus subtilis* (PTCC1715).

							Erythromycin concentration
Concentration μg/mL shell	10,000	5,000	2,500	1,250	625	312	32 μg/mL
*E. coli*	−	−	−	−	−	+	−
*B. subtilis*	−	−	+	+	+	+	−
Concentration μg/mL stem	10,000	5,000	2,500	1,250	625	312	32 μg/mL
*E. coli*	−	−	−	−	−	+	−
*B. subtilis*	−	−	+	+	+	+	−
Concentration μg/mL moss	10,000	5,000	2,500	1,250	625	312	32 μg/mL
*E. coli*	−	−	−	−	−	−	−
*B. subtilis*	−	−	+	+	+	+	−
Concentration μg/mL shell	8,000	4,000	2,000	1,000	500	250	32 μg/mL
*E. coli*	−	−	−	−	+	+	−
*B. subtilis*	−	−	+	+	+	+	−
Concentration μg/mL stem	8,000	4,000	2,000	1,000	500	250	32 μg/mL
*E. coli*	−	−	−	−	+	+	−
*B. subtilis*	_	−	+	+	+	+	−
Concentration μg/mL moss	8,000	4,000	2,000	1,000	500	250	32 μg/mL
*E. coli*	−	−	−	−	−	+	−
*B. subtilis*	−	−	+	+	+	+	−
Concentration μg/mL shell	6,000	3,000	1,500	750	375	182	32 mL/ μg
*E. coli*	−	−	−	−	+	+	−
*B. subtilis*	−	+	+	+	+	+	−
Concentration μg/mL stem	6,000	3,000	1,500	750	375	182	32 μg/mL
*E. coli*	−	−	−	−	+	+	−
*B. subtilis*	_	+	+	+	+	+	−
Concentration μg/mL moss	6,000	3,000	1,500	750	375	182	32 μg/mL
*E. coli*	−	−	−	−	−	+	−
*B. subtilis*	_	−	+	+	+	+	−

The same was reported for *B. subtilis* and MIC between 8 and 20 mg/mL were declared (Keypour et al., 2008; Quereshi et al., 2010; Mehta and Jandaik, 2012). Surprisingly, the culture conditions attained through the statistical method stimulated bioactive compounds with valuable antimicrobial activity in the fruiting body. Therefore, the highest antimicrobial activity compared to the previous research was obtained in this research. Consequently, antimicrobial compounds from *G. lucidum* moss could be proposed as good therapeutic potential.

### Cytotoxicity *of Ganoderma lucidum* against human breast cancer cell line MDA-MB-231

**Figure 10:**
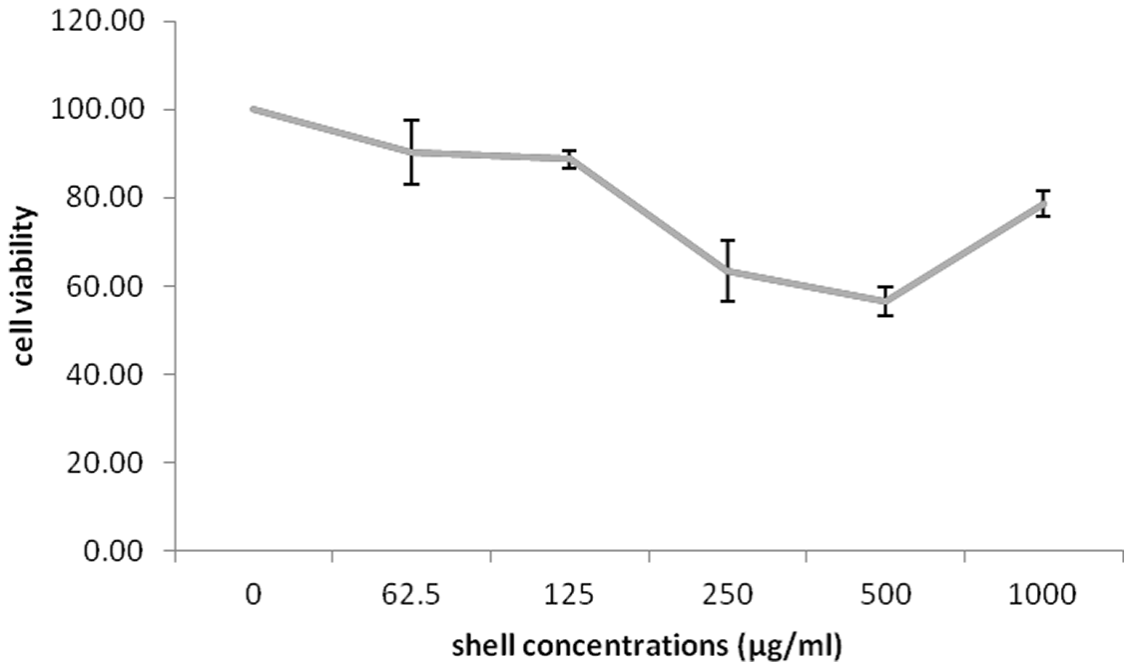
Cytotoxic impact of *Ganoderma lucidum* shell on MDA-MB-231 cells. Each bar indicated the mean ± SD of three experiments.

**Figure 11:**
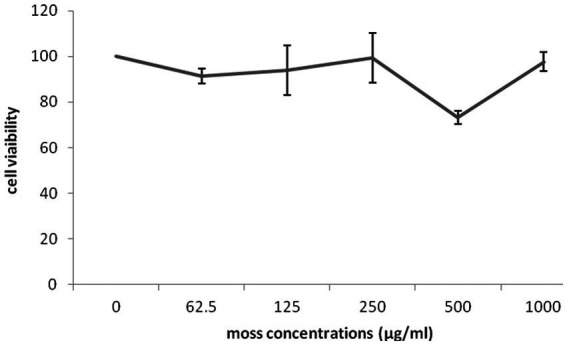
Cytotoxic impact of *Ganoderma lucidum* moss on MDA-MB-231 cells. Each bar indicated the mean ± SD of three experiments.

**Figure 12:**
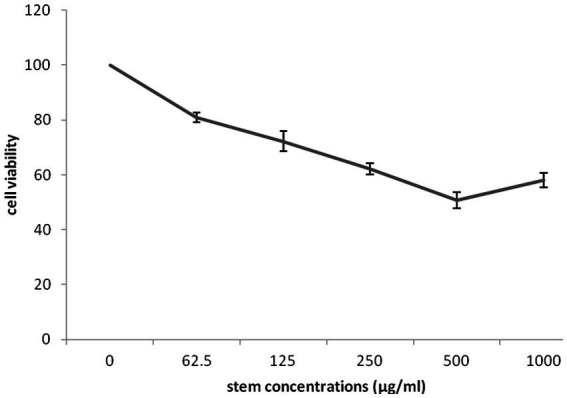
Cytotoxic impact of *Ganoderma lucidum* stem on MDA-MB-231 cells. Each bar represented the mean ± SD of three experiments.

The impact of different concentrations (0–1,000 μg/mL) of different part of *G. lucidum* on the growth of highly invasive, estrogen-independent (MDA-MB-231) breast cancer cells was evaluated in a dose dependent manner for 48 h. Although extensive research has been carried out on antitumor potential of *G. lucidum* extract (Jiang et al., 2004, 2006; de Camargo et al., 2022), What is still unclear is the impact of extracting different parts of the fruiting body of *G. lucidum* in against breast cancer cell line MDA-MB-231. MTT assay was performed to evaluate the cytotoxicity capacity of *G. lucidum* shell, stem, and, moss against MDA-MB-231 cells. This study delved into the potential anti-cancer benefits of different parts of the *Ganoderma* mushroom fruit for the first time. The result indicated that the stem and shell of *G. lucidum* significantly aggravated cell death at a dose of 500 μg/mL. The IC50 levels of shell and stem of *G. lucidum* were found to be around 465.3 and 485.7 μg/mL, for 48 h incubation while moss did not reach IC50 levels at inhibitor concentrations (Figures 10, 11, 12).

To date, more than 300 bioactive compounds have been isolated from various parts of *G. lucidum*, including peptides, fatty acids, polysaccharides, and especially triterpenoids (Sharma et al., 2019). Studies indicate that different extracts or isolated compounds from *G. lucidum* act as a carcinostatic on different cancer cell lines, such as, lung (Li et al., 2013), colon (Li et al., 2020), pancreas (Chen et al., 2023), liver (Li et al., 2005), breast (Barbieri et al., 2017), skin (Shahid et al., 2022), and prostate (Wang et al., 2020). Among these compounds, triterpenoids are the most important bioactive compounds of *G. lucidum*, whose inhibitory effects on many cancers have been widely studied (Zhao et al., 2023). In this research, glucose as a carbon substrate and CSL syrup as a protein substrate was rated as the best substrates for stimulating an important biological compound, namely ganoderic acids. These optimal conditions led to an increased in the amount of the biologically active compound in the *G. lucidum* fruit. So that stem and shell of *G. lucidum* showed a significant control effect on MDA-MB-231 cells.

## Conclusion

The objectives of this research are to find out the effect of the key substrates and their concentrations on CGLP and GA productions using OFAT as screening method prior to RSM. The highest content of GA in TiO_2_ NPs with the highest content of glucose syrups and CSL syrups indicated that biosynthesis of GA in *G. lucidum* was triggered by chemical stressors. The moss of *G. lucidum* fruit-body with a wide variety of flavonoids and phenolic acids had the highest antimicrobial activity against *E. coli* and *B. subtilis*. The shell of *G. lucidum* induced the most prominent cytotoxicity against the MDA-MB-231 cell line with the lowest IC50. In conclusion, the optimized methods employed enhanced the quality and quantity of the bioactive components of the shell and moss of *G. lucidum* fruit body with the highest prominent cytotoxicity against cancer cells and significant antimicrobial activity, respectively, and probably performs the same way for the other medical mushroom.

## Data availability statement

The original contributions presented in the study are included in the article; further inquiries can be directed to the corresponding author.

## Ethics statement

This is an observational study. The XYZ Research Ethics Committee has confirmed that no ethical approval is required.

## Author contributions

AT: Data curation, Formal analysis, Investigation, Project administration, Resources, Software, Writing – review & editing. HS: Conceptualization, Data curation, Formal analysis, Investigation, Methodology, Project administration, Software, Supervision, Validation, Visualization, Writing – review & editing. AS: Conceptualization, Data curation, Formal analysis, Investigation, Methodology, Project administration, Software, Supervision, Validation, Visualization, Writing – review & editing. AG: Data curation, Investigation, Project administration, Resources, Writing – review & editing.
